# Transfer language space with similar domain adaptation: a case study with hepatocellular carcinoma

**DOI:** 10.1186/s13326-022-00262-8

**Published:** 2022-02-23

**Authors:** Amara Tariq, Omar Kallas, Patricia Balthazar, Scott Jeffery Lee, Terry Desser, Daniel Rubin, Judy Wawira Gichoya, Imon Banerjee

**Affiliations:** 1 Machine Intelligence in Medicine and Imaging (MI ∙2) Lab, Mayo Clinic, Phoenix, AZ USA; 2 Department of Radiology, Emory University, Atlanta, GA USA; 3 Department of Radiology, Stanford University, Palo Alto, CA USA; 4 Department of Biomedical Data Science, Stanford University, Palo Alto, CA USA

**Keywords:** Transfer learning, Language model, Radiology report, BERT, Word2vec

## Abstract

**Background:**

Transfer learning is a common practice in image classification with deep learning where the available data is often limited for training a complex model with millions of parameters. However, transferring language models requires special attention since cross-domain vocabularies (e.g. between two different modalities MR and US) do not always overlap as the pixel intensity range overlaps mostly for images.

**Method:**

We present a concept of similar domain adaptation where we transfer inter-institutional language models (context-dependent and context-independent) between two different modalities (ultrasound and MRI) to capture liver abnormalities.

**Results:**

We use MR and US screening exam reports for hepatocellular carcinoma as the use-case and apply the transfer language space strategy to automatically label imaging exams with and without structured template with > 0.9 average f1-score.

**Conclusion:**

We conclude that transfer learning along with fine-tuning the discriminative model is often more effective for performing shared targeted tasks than the training for a language space from scratch.

## Introduction

Hepatocellular carcinoma (HCC) is the most common primary liver malignancy, and is the fastest-rising cause of cancer-related deaths in the United States [1]. Imaging surveillance for HCC is recommended in high-risk patients, which includes those with cirrhosis and/or chronic hepatitis B viral infection [2]. The goal of imaging surveillance is the early detection of HCC in these patients, while they are still within a curative window [3]. Multiple imaging modalities are used for HCC screening and diagnosis; these include ultrasound (US), contrast-enhanced ultrasound, CT and Magnetic Resonance (MR) [2, 3]. There is significant variation in HCC screening protocols across various institutions; and as a result, patients may receive a mix of imaging studies (US, CT, MRI) across their longitudinal screening record.

The Liver Imaging Reporting and Data System (LI-RADS) was developed by the American College of Radiology (ACR) as a standardized coding system for HCC imaging surveillance [4]. The LI-RADS system utilizes imaging features to categorize liver lesions based on risk of malignancy, while standardizing report terminology. Different versions of LI-RADS are available for each of the HCC screening imaging modalities (including US and MRI). Although the LI-RADS categories within each system are similar, the different versions account for differences in modality-specific lexicon. LI-RADS standardized reporting systems, and the use of structured HCC-screening imaging report templates, help facilitate information extraction from imaging reports, enabling the creation of large-scale annotated databases that can be used for clinical outcomes and machine learning research. Despite these efforts, adoption of structured reports remains low [5], and simple tasks like differentiating between benign and malignant exams from a large number of screening cases, requires hundreds of hours of manual review by the radiologist. In our institution, which is a large multi-site academic center, structured reports coded with LI-RADS were adopted recently in 2018, representing only a small fraction of available HCC screening data.

Natural language processing (NLP) has been utilized to extract information from, and classify imaging reports following standard guidelines [6, 7]. Traditional NLP methodology includes rule-based, dictionary-based and supervised learning [7–10] techniques. A major limitation of these techniques is the requirement for large-scale human-labeled data; or explicit linguistic rules that would limit the scalability and generalizability of the system. Additionally, the use of experts to perform the required medical data annotation is expensive and time-consuming; thereby limiting data cohort size. In this paper, we explore recent language modeling methodologies that overcome these limitations: (1) context-independent word embedding models (Word2vec [11], Glove [12]), where the language model (LM) learns numerical representation of words regardless of where the word occurs in a sentence, and (2) context-dependent word embedding models (BERT [13], ELMo [14]), which capture the context of words – that is, it’s position in a sentence.

In our previous work, we developed a context-independent word embedding-based NLP pipeline [15], that can infer binary LI-RADS categories from the liver section of HCC screening US reports. Only a minimal amount of human-labeled data was required to train this model. Our healthcare institutions recently adopted the structured LI-RADS reporting templates for HCC-screening using MR and have limited data available with structured reporting; therefore, fine-tuning language models may allowed to overcome the challenge of labeled data scarcity and helps to annotate imaging exams coded without LI-RADS template.

**Fig. 1:**
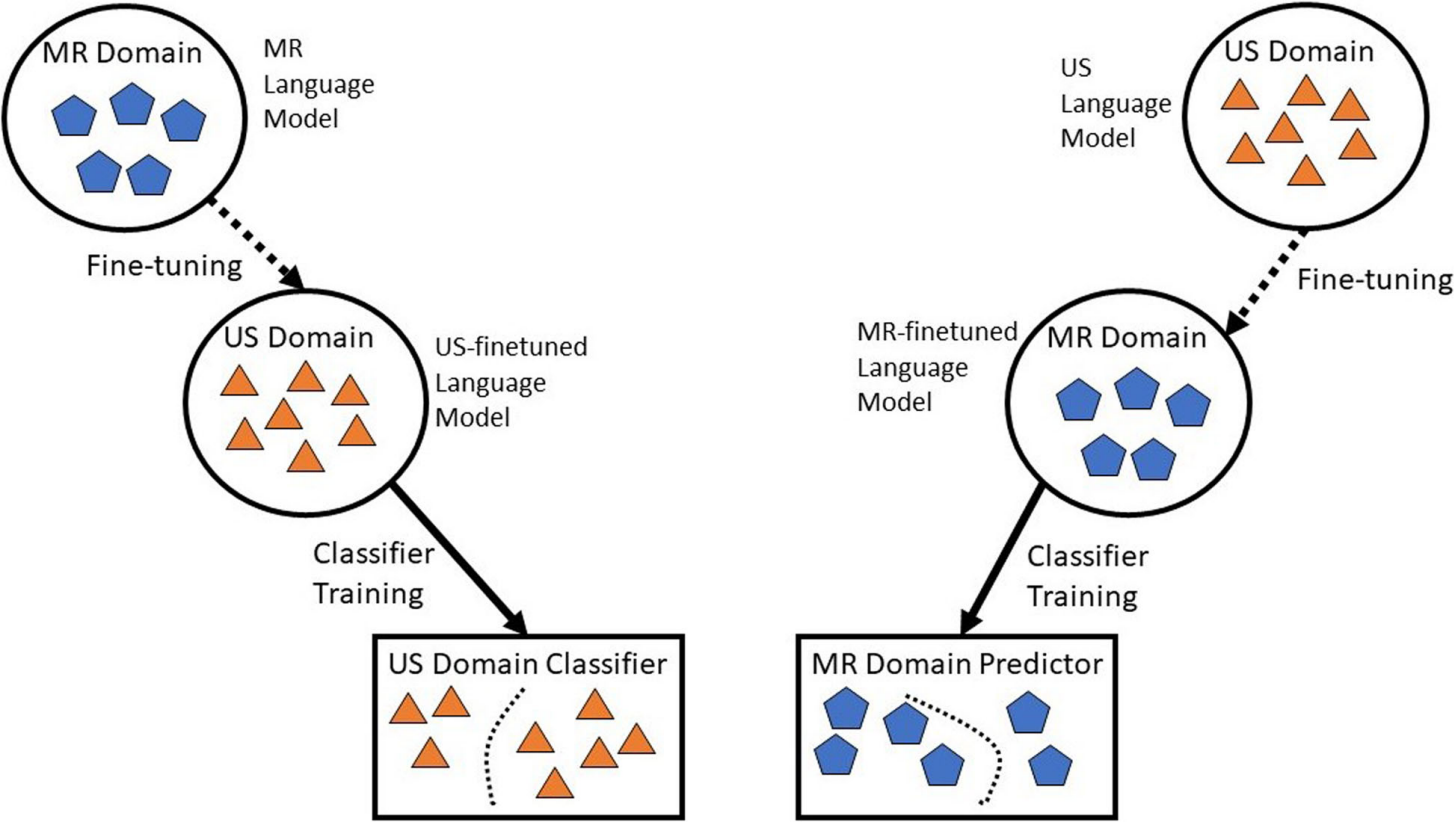
Cross domain finetuning language modeling schemes for MR and US domains

In this paper, we developed both *context-dependent* and *context-independent* language models (LM) trained on US reports from one institution, Stanford Health Care (SHC), to infer the labels from MR studies from another institution, Emory University Healthcare (EUH); and vice-versa. Figure 1 summarizes our modeling scheme for cross-domain fine-tuning. We worked with two language domains, i.e., MR and US domain, which share common HCC screening terminology, but differ in modality-specific lexicon. The language model trained over the MR domain was fine-tuned over the US domain and then used to train a classifier for the US domain. Similarly, the language model trained over US domain was fine-tuned over the MR domain, and then used to train a classifier for MR domain. To generate a large dataset for HCC screening, the aim of the study was to label the all free-text reports obtained between 2010 – 2017 in Stanford and Emory without the LI-RADS formatted template. To demonstrate the benefit of similar domain adaptation, we compare the performance of the fine-tuned LM with the directly trained language models. In addition, we compare the performance of context dependent and context-independent language models by integrating with different discriminative model combinations (RandomForest, LSTM, 1DCNN).

## Method

### Datasets

#### Stanford US dataset

With the approval of Stanford Institutional Review Board (IRB), we collected all the free-text radiology reports of abdominal ultrasound examinations performed at Stanford Hospital between August 2007 to December 2017. In total, there were 92,848 US reports collected over 10 years, with an average 9,000 US exams performed every year. Among them, 13,860 exams were performed for HCC screening. A total 1,744 abdominal US studies were coded with the US LI-RADS reporting format where a unique LIRADS score was reported in the impression section.

#### EUH MRI dataset

**Fig. 2:**
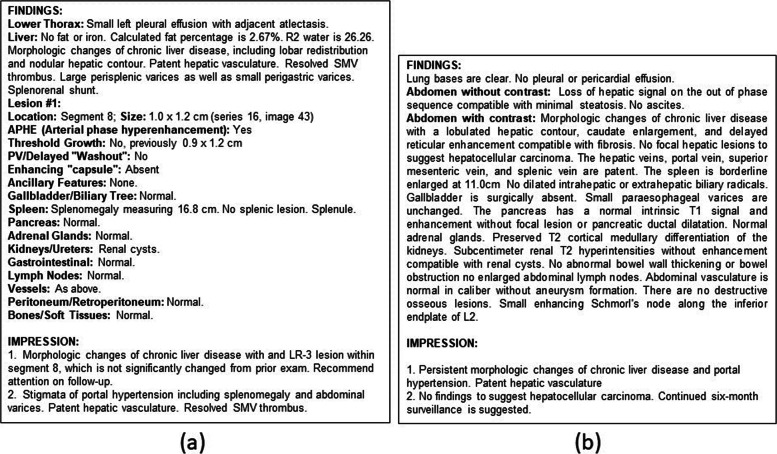
Sample MR reports: (**a**) Sample with LI-RADS structured template, (**b**) Sample free-text report

With the approval of Emory University IRB, we retrieved 10,018 MRI exams performed between 2010 - 2019 at EUH for HCC screening. Among these, only 548 studies were reported using the LI-RADS structured template where a unique LI-RADS score was reported in the impression section (Fig. 2a). From the LI-RADS coded reports, 99% were malignant cases (LR score > 2) since benign cases are often not coded with LI-RADS. 9,470 MRI abdomen exams were documented as free-text narratives where the final diagnosis was recorded without following any structured scoring schema (Fig. 2b). To obtain a representative sample of benign cases from the MR studies (which represented 1% of the LI-RADS coded reports), two radiologists manually annotated 537 benign cases from the EUH MRI dataset. We selected benign cases from reports performed after 2018 in order to match the report structure of annotated malignant cases.

#### Synopsis of the datasets

In Table 1, we present the synopsis of the Stanford US dataset and EUH MRI dataset according to report-level and word-level statistics which reflects a slight diversity between the style of reporting. For instance, the number of words in the reports ranged from 24 to 331 in the US dataset while for MR dataset in varies from 11 to 2116. Same observation holds for the number of sentences in the reports. It is also interesting to note that there were 1790 common words between MR and US.

**Table 1:** Statistics of the cohorts before processing - Stanford US dataset and EUH MRI dataset

	Stanford US dataset	EUH MRI dataset
**Cohort level**		
Number of unique words	17194	19828
Common words in two domains	1790	
**Report level**		
Average number of words (+/- std)	167 (+/- 39)	197 (+/-47)
Average number of sentences (+/- std)	27 (+/-7)	32 ((+/-8)
Number of unique words in templated reports		2774
Number of unique words in reports without template		7930
Average number of words (+/- std) describing		
liver related finding in templated reports	36 (+/- 25)	109 (+/- 63)
Average number of words (+/- std) describing		
liver related finding in reports without template	47 (+/- 27)	104 (+/- 52)

#### Annotated datasets

We evaluated the efficiency of our transfer learning scheme on the task of HCC malignancy scoring. We have malignancy information available for the following sets of reports.

i) Templated US reports from Stanford dataset: These reports are associated with LI-RADS scores. LI-RADS > 2 is not definitely benign. We experimented with a collection of 1462 reports which are split in training and test sets. Test sets contains 29 reports with ‘malignant’ label and 264 reports with ‘benign’ label.

ii) Templated MR reports from EUH MRI dataset: We have a total of 944 MR reports with associated LI-RADS scores such that LI-RADS > 2 indicates that the lesion is not definitely benign. This set is split into training and test sets. Test sets contains 81 reports with ‘malignant’ label and 108 reports with ‘benign’ label.

iii) US reports without template from Stanford dataset: We randomly sampled 142 US reports and two expert radiologists associated each selected report with a LI-RADS score (Cohen kappa 0.85). 11 reports were labelled ‘malignant’ while the remaining 131 reports were labelled as ‘benign’. Malignancy prediction model trained on structured US reports is tested over these unstructured reports.

iv) MR reports without template from EUR MRI dataset: We randomly sampled 112 unstructured MR reports with no LI-RADS scores. Two expert radiologist assigned LI-RADS scores for these reports with malignancy indicated by LI-RADS > 2 (Cohen kappa 0.92). 21 reports were labelled ‘malignant’ while the remaining 91 reports were labelled as ‘benign’. We test our malignancy prediction model trained over structured MR reports on these sampled unstructured reports.

### Report pre-processing

#### Segmentation

We design a python-based liver section segmentation module that works with both MRI/CT and US reports. The model uses a combination of regular expressions and dictionary based sentence retrieval using anatomical vocabularies derived from Foundational Model of Anatomy (FMA) [16] to extract only findings related to liver and its sub-regions from the whole report. The module maintains dependencies between anatomical entities (e.g. all the lesions found within the liver would be extracted even if they are not described in the same paragraph). This segmentation module has been manually validated on randomly selected 100 MRI and 100 US reports and obtained perfect accuracy for segmenting the liver section and liver related statements from both recent (more structured) and historic radiology reports. In order to perform a valid experiment from the LI-RADS formatted US and MRI reports, we excluded the Impression section of the reports since the final LI-RADS assessment category is often reported explicitly in the Impression. The Findings section includes only the imaging characteristics of the liver abnormalities; thus, it does not contain any clear-cut definition of the LI-RADS final assessment classes.

#### Text cleaning

We normalize the text by converting in to lowercase letters, removing general stop words (e.g. ‘a’, ‘an’, ‘are’, ‘be’, ‘by’, ‘has’, ‘he’, etc.), removing words with very low frequency (< 50). We also removed unwanted terms/phrases (e.g. medicolegal phrases such as “I have personally reviewed the images for this examination”); these words generally appear in all or very few reports, and are thus of little to no value in document classification. We used the Natural Language Tool Kit (NLTK) library [17] for determining the stop-word list and discarded these words during report indexing. We also discarded radiologist, clinician, and patient-identifying details from the reports. Date and time stamps were replaced by ‘ <*datetoken*>’ phase.

### Language modeling

Language modeling allows for learning vector representation of text such that semantic characteristics of the text are preserved. We were able to use both labeled and unlabelled US and MR reports for training language models for US and MR domains since training the language models does not need supervised labels. We used the following two approaches for language modeling - context-dependent (BERT) and context-independent (Word2Vec) language modeling.

#### Word2Vector language model

Word2vec language modeling schemes captures finer semantic properties of words by learning a vector representation corresponding to each word. Instead of one-to-one mapping between a word and an element in the vector, representation is distributed across several hundred dimensions, mapping to words to a new representation space such that semantically similar words are mapped closer to each other than semantically dissimilar words. Our model learns such vector representation by training a skipgram model with hierarchical softmax [11]. Since training such models require no labels, LI-RADS scores are not needed for language model training.

We trained two base word2vec models; i) US word2vec, and ii) MR word2vec. US word2vec language model was trained using all US reports from Stanford US dataset regardless of availability of LI-RADS scores. MR word2vec model was trained using all MR reports from EUH MRI dataset regardless of availability of LI-RADS scores.

We also trained two cross-domain language models using transfer learning; ii) US-finetuned word2vec, and ii) MR-finetuned word2vec. For US-finetuned word2vec, words of the US domain that are common with MR domain are initialized by MR word2vec vectors. The model is further finetuned on US reports. Similarly, common words are initialized by using US word2vec vectors for MR-finetuned word2vec model that is further finetuned on MR reports from EUH MRI dataset. These models do not have to learn from scratch. Instead, they can take advantage of language model training performed in a separate but similar domain.

#### BERT language model

BERT learns bi-directional representations for text by jointly conditioning on both left and right context [13]. We used BERT to train a masked language model that is optimized to be able to predict masked tokens from the sentences. We trained two language models using BERT; i) US BERT - by training on all US reports from Stanford dataset, and ii) MR BERT - by training all MR reports from EUH MRI dataset. Similar to word2vec modeling, we also trained two cross-domain language model using transfer learning for BERT as well; iii) US-finetuned BERT, and iv) MR-finetuned BERT. For US-finetuned BERT, common words of US and MR domains are initialized by MR BERT vectors and the model is finetuned on US reports. Similarly, common words are initialized by using US BERT vectors for MR-finetuned BERT model that is further finetuned on MR reports from EUH MR dataset. BERT models are usually limited in terms of length of input text sequence they can process. We employ only portion of the report discussing liver as input to all our models. Thus, our models generally work with smaller length text sequences. Wherever needed, text is clipped to fit into BERT model.

### Predictive modeling

We experimented with the following three predictive models.

i) Language model vectors + RF classifier: In this setup, we use our language models to generate embeddings for input reports. We then train a discriminative model - Random Forest (RF) binary classifier to predict ‘malignant’ or ‘benign’ labels for each input report.

ii) 1D-CNN model: Preserving context is one of the prominent differences between BERT and Word2vec. Thus we applied one-dimensional convolutional neural network (1D-CNN) with random word embedding generated that uses one-dimensional convolutional filters to process textual sequences and can learn important semantic structured such as phrases using, while avoiding memorization of entire text sequence. The model consists of *Embedding* layer, one-dimensional *Convolutional* layer, *Dropout* and *Dense* layers.

iii) Word2vec embedding + 1D-CNN: Normally, weights of *Embedding* layer in 1D-CNN are initialized randomly as weights of all other layers, and then finetuned over training input-output tuples. We designed a separate classifier by initializing *Embedding* layer of 1D-CNN with the weights of word2vec model. While the overall classifier is still trained over input-output tuples of training data, *Embedding* layer is able to take advantage of unlabelled data as well as word2vec models are trained over both labelled and unlabelled reports.

### Experimental setup

We used the following four experimental setups to thoroughly evaluate the advantages of our transfer learning scheme for the language space.

**Setting 1) - MR language model, tested on MR:** Under this setting, language model is trained over MR reports. Trained language model is used to generate feature vectors for MR reports with and without template.Vectorized reports with template are used to train the classifier. The trained classifier is then used to evaluate test sets consisting of MR reports with template and MR reports without template. Since we are working with multiple language models and classifiers, the following experiment titles fall under this setting; i) ‘MR Word2Vec+Random Forest Classifier’, ii) ‘MR Word2Vec Embedding+1DCNN’, iii) ‘MR BERT+Random Forest Classifier’.

**Setting 2) - MR-finetuned language model, tested on MR:** Under this setting, language model trained over US reports is used to initialize vectors for common terms between US and MR domains. Such initialized language mode is further fine-tuned over MR reports to generate vectors for all MR terms. Finetuned language model is used to generate feature vectors for MR reports with and without template. The same classifier training and testing process is applied as described for setting 1. The following experiment titles fall under this setting; i) ‘MR-finetuned Word2Vec+Random Forest Classifier’, ii) ‘MR-finetuned Word2Vec Embedding+1DCNN’, iii) ‘MR-finetuned BERT+Random Forest Classifier’.

**Setting 3) - US language model, tested on US:** Under this setting, language model is trained over US reports. Feature vectors for all structured (with template) and unstructured (without template) US reports are generated using this trained language model. Feature vectors of reports from the training set of US reports with template are used to train chosen classifier to detect malignancy. The trained classifier is then used to evaluate test sets consisting of US reports with template and US reports without template. The following experiment titles fall under this setting; i) ‘US Word2Vec+Random Forest Classifier’, ii) ‘US Word2Vec Embedding+1DCNN’, iii) ‘US BERT+Random Forest Classifier’.

**Setting 4) - US-finetuned language model, tested on US:** Under this setting, language model trained over MR reports is used to initialize vectors for common terms between US and MR domains. Such initialized language mode is further finetuned over US reports to generate vectors for all US terms. Finetuned language model is used to generate feature vectors for US reports with and without template. The same classifier training and testing process is applied as described for setting 3). The following experiment titles fall under this setting; i) ‘US-finetuned Word2Vec+Random Forest Classifier’, ii) ‘US-finetuned Word2Vec Embedding+1DCNN’, iii) ‘US-finetuned BERT+Random Forest Classifier’.

Experiment titles in the Results section are consistent with the terminology presented above for clarity. Note that under all of these settings, classifier is trained only using reports with template while it is tested over two test sets; one consisting of reports with template, and one consisting of reports without template.

## Results

### Performance analysis

**Fig. 3:**
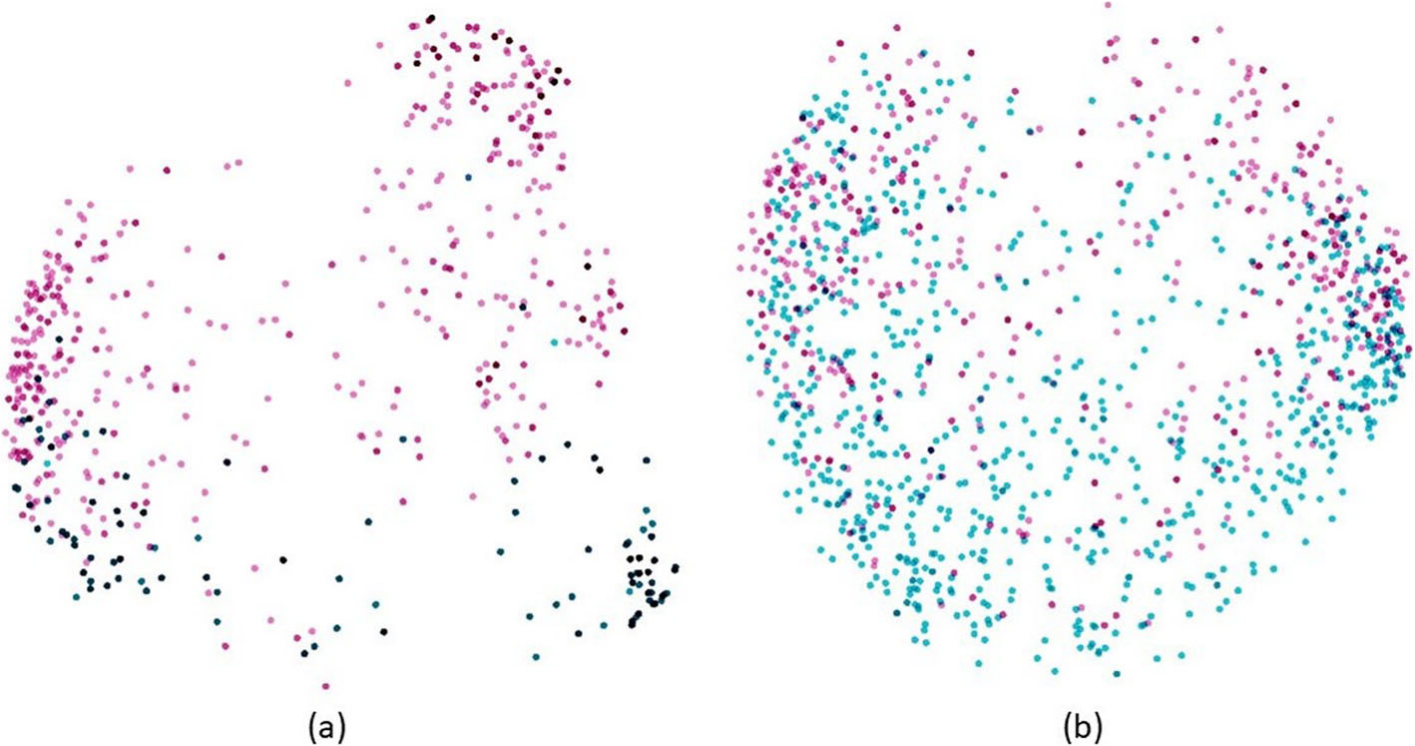
Sample reports: (**a**) Embedding of structured and unstructured US reports structured template, (**b**) Embedding of structured and unstructured MR reports structured template

Figure 3 presents the 2D embedding of MR and US reports which shows that structured/templates and unstructured/untemplated reports retains significant differences in the language space, given the variations in syntax. Thus it is a challenging task to infer labels on the unstructured reports using a model only trained on labeled reports. In Tables 2 and 3, we present the results of our predictive models as described in the *Experimental Setup*, on both structured and unstructured MR and US reports respectively. It is clear from Table 2 that fine-tuned language models perform better when paired with any of the selected classifiers for the more challenging task of classifying MR reports without template with highest overall weighted f1-score 0.90. This trend is true for both language models (Word2Vec and BERT) as well as both classifiers (Random Forest and 1DCNN). The biggest performance difference with cross-domain fine-tuning is observed between ‘MR-finetuned Word2Vec Embedding+1DCNN’ with weighted f1-score 0.87 and ‘MR Word2Vec Embedding+1DCNN’ with f1-score 0.76. This difference can be attributed to the fact that we allowed both language model Word2Vec and then the 1DCNN weights to be finetuned. All other experiments allow for only fine-tuning of the language model. For structured report, models with and without finetuning tend to perform similarly.

**Table 2:** Performance of language model and classifiers on structured MR reports (reports with template) and unstructured MR reports (reports without template). Models trained over MR domain as well as cross-domain models (MR-finetuned) have been tested

	Report document with template			Report document without template		
	Precision	Recall	f1-score	Precision	Recall	f1-score
	**MR-finetuned Word2Vec+Random Forest Classifier**					
*Malignant*	0.94	0.90	0.92	0.73	0.76	0.74
*Benign*	0.93	0.95	0.94	0.94	0.93	0.94
	**MR Word2Vec+Random Forest Classifier**					
*Malignant*	0.95	0.93	0.94	0.70	0.76	0.73
*Benign*	0.95	0.96	0.95	0.94	0.92	0.93
	**MR-finetuned Word2Vec Embedding+1DCNN**					
*Malignant*	0.95	0.98	0.96	0.68	0.62	0.65
*Benign*	0.98	0.96	0.97	0.91	0.93	0.92
	**MR Word2Vec Embedding+1DCNN**					
*Malignant*	0.97	0.96	0.97	0.40	0.19	0.26
*Benign*	0.97	0.98	0.98	0.83	0.93	0.88
	**MR-finetuned BERT+Random Forest Classifier**					
*Malignant*	0.94	0.90	0.92	0.71	0.57	0.63
*Benign*	0.93	0.95	0.94	0.91	0.95	0.92
	**MR BERT+Random Forest Classifier**					
*Malignant*	0.94	0.91	0.92	0.77	0.48	0.59
*Benign*	0.94	0.95	0.94	0.89	0.97	0.93

**Table 3:** Performance of language model and classifiers on structured US reports (reports with template) and unstructured US reports (reports without template). Models trained over US domain as well as cross-domain models (US-finetuned) have been tested

	Report document with template			Report document without template		
	Precision	Recall	f1-score	Precision	Recall	f1-score
	**US-finetuned Word2Vec+Random Forest Classifier**					
*Malignant*	0.68	0.52	0.59	0.75	0.27	0.40
*Benign*	0.95	0.97	0.96	0.94	0.99	0.97
	**US Word2Vec+Random Forest Classifier**					
*Malignant*	0.71	0.59	0.64	0.67	0.18	0.29
*Benign*	0.96	0.97	0.96	0.94	0.99	0.96
	**US-finetuned Word2Vec Embedding+1DCNN**					
*Malignant*	0.68	0.66	0.67	0.70	0.64	0.67
*Benign*	0.96	0.97	0.96	0.97	0.98	0.97
	**US Word2Vec Embedding+1DCNN**					
*Malignant*	0.68	0.72	0.70	0.64	0.64	0.64
*Benign*	0.97	0.96	0.97	0.97	0.97	0.97
	**US-finetuned BERT+Random Forest Classifier**					
*Malignant*	0.91	0.34	0.50	0.33	0.09	0.14
*Benign*	0.93	1.00	0.96	0.93	0.98	0.96
	**US BERT+Random Forest Classifier**					
*Malignant*	0.67	0.34	0.45	0.33	0.09	0.14
*Benign*	0.93	0.98	0.96	0.93	0.98	0.96

Table 3 which reports the performance on the US reports, shows a similar trend as Table 2. US-finetuned language models (best weighted f1-score 0.95) perform better when paired with any of the classifiers (Random Forest or 1DCNN) for the more challenging task of classifying US reports without template. This trend highlights the fact that cross-domain fine-tuning of the language space helps to improve the performance.

An interesting trend is observed while comparing Word2Vec and BERT language models, paired with Random Forest classifier in both Tables 2 and 3. Even though BERT language model is supposed to generate a contextual representation of tokens taking into account words sequence, it performs worse than simple Word2Vec model with 3% drop in the overall weighted f1-score. This trend can be attributed to the fact that proper training of end-to-end BERT model requires large training data which is not available in either domain in our experiments. Thus, we combined BERT masked model with classifiers to train on few thousand documents only.

### Visualization

**Fig. 4:**
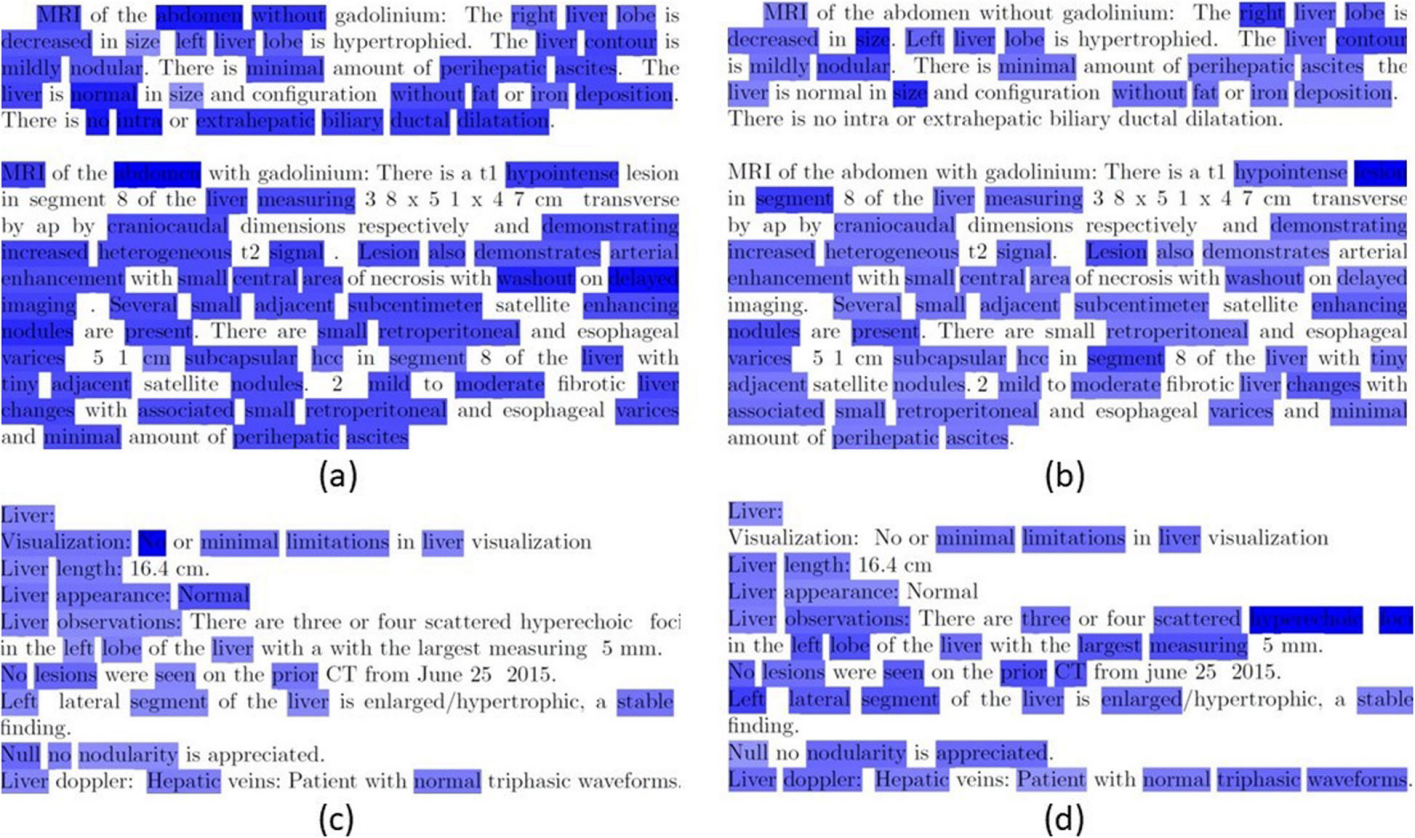
Heatmap of liver-related text of sample MR and US reports with ‘Malignant’ label: (**a**) MR Word2Vec Embedding+1DCNN - predicted label: ‘Benign’, (**b**) MR-finetuned Word2Vec Embedding+1DCNN - predicted label: ‘Malignant’,(**c**) US Word2Vec Embedding+1DCNN - predicted label: ‘Benign’, (**d**) US-finetuned Word2Vec Embedding+1DCNN - predicted label: ‘Malignant’

We adopted visualization schemes to explore the inner workings of our language model. We visualized importance of each word in the input text to our 1D-CNN model by generating heatmaps using sensitivity analysis [18]. Such analysis relies on partial derivative of the loss function with respect to each word of the text. Figure 4 shows heatmaps of one randomly selected reports with and without fine-tunned language model. Intensity of blue color indicates the weight given to each word for this prediction, higher weights indicates darker color. Figure 4-a shows heatmap for 1D-CNN with word2vec MR language model. This model mis-predicts the class label as ‘Benign’ while actually this text comes from a report associated with a ‘Malignant’ study. The model is highly focused on the sentence ‘There is no intra or extrahepatic biliary ductal dilatation’ in addition to all sentences explaining problem with the liver. This is probably the reason for mis-classification as ‘Benign’. Figure 4-b shows the heatmap for the same text from 1D-CNN model with MR-finetuned word2vec language model. This model is able to focus on the correct section of the report explaining reasons for malignancy. This model focuses on many sentences listing liver problems and ignores the sentence stating normalcy of ductal dilation. Hence, it predicts correct class label, i.e., ‘Malignant’. Similar examples are shown in Figs. 4-c and 4-d for a US reports with US word2vec embedding and US-finetuned word2vec embedding, respectively, with 1DCNN classifier. Classifier is unable to focus on ‘hyperechoic foci’ when US word2vec embedding is used, results in incorrect prediction of ‘benign’ label. This mis-prediction is corrected by the same 1DCNN architecture when US-finetuned word2vec embedding is used.

**Fig. 5:**
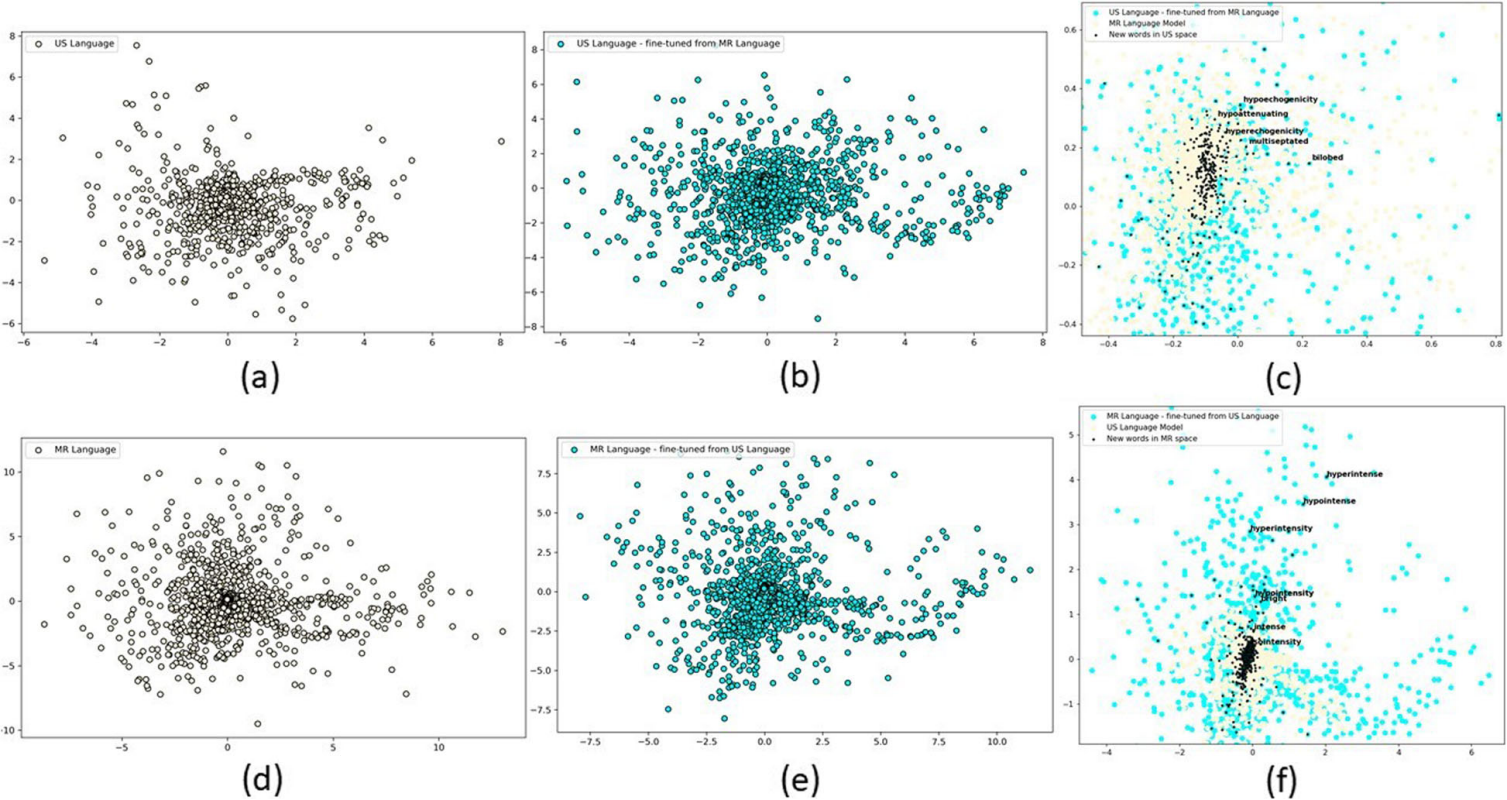
Word2Vec Language Spaces; (**a**): US Language Model, (**b**): US-finetuned Language Model, **c**): New words in US-finetuned Language Model, (**d**): MR Language Model, (**e**): MR-finetuned Language Model, (**f**): New words in MR-finetuned Language Model

Figure 5 show our visualization of word2vec language models where we individually plotted US word2vec, MR word2vec, US-finetuned word2vec, MR-finetuned word2vec after reducing the dimension of embedding using t-SNE. In addition, we have plotted overlapping plot for US word2vec and MR-finetuned word2vec which initializes common words from US word2vec. New words in MR-finetuned word2vec have been marked. Zoomed-in image clearly shows that semantically similar words (e.g., ‘isointensity’, ‘hypointensity’, ‘hyperintensity’, ‘intense’, ‘bright’, ‘hypointense’, ‘hyperintense’,‘hypointensity’) in the original and finetunned language space are mapped close together. Similar plot has been generated for MR word2vec, US-finetuned word2vec and new words in US-finetuned word2vec. Similar observation can be made about this plot. Words semantically related to ‘echoic’ or reflective characteristics (‘hypoechogenicity’, ‘hyperechogenicity’, ‘hypoattenuating’, ‘hypoechoic’, ‘hyperechoic’), and shape-related words like ‘multiseptated’ and ‘bilobed’ are mapped close together.

**Fig. 6:**
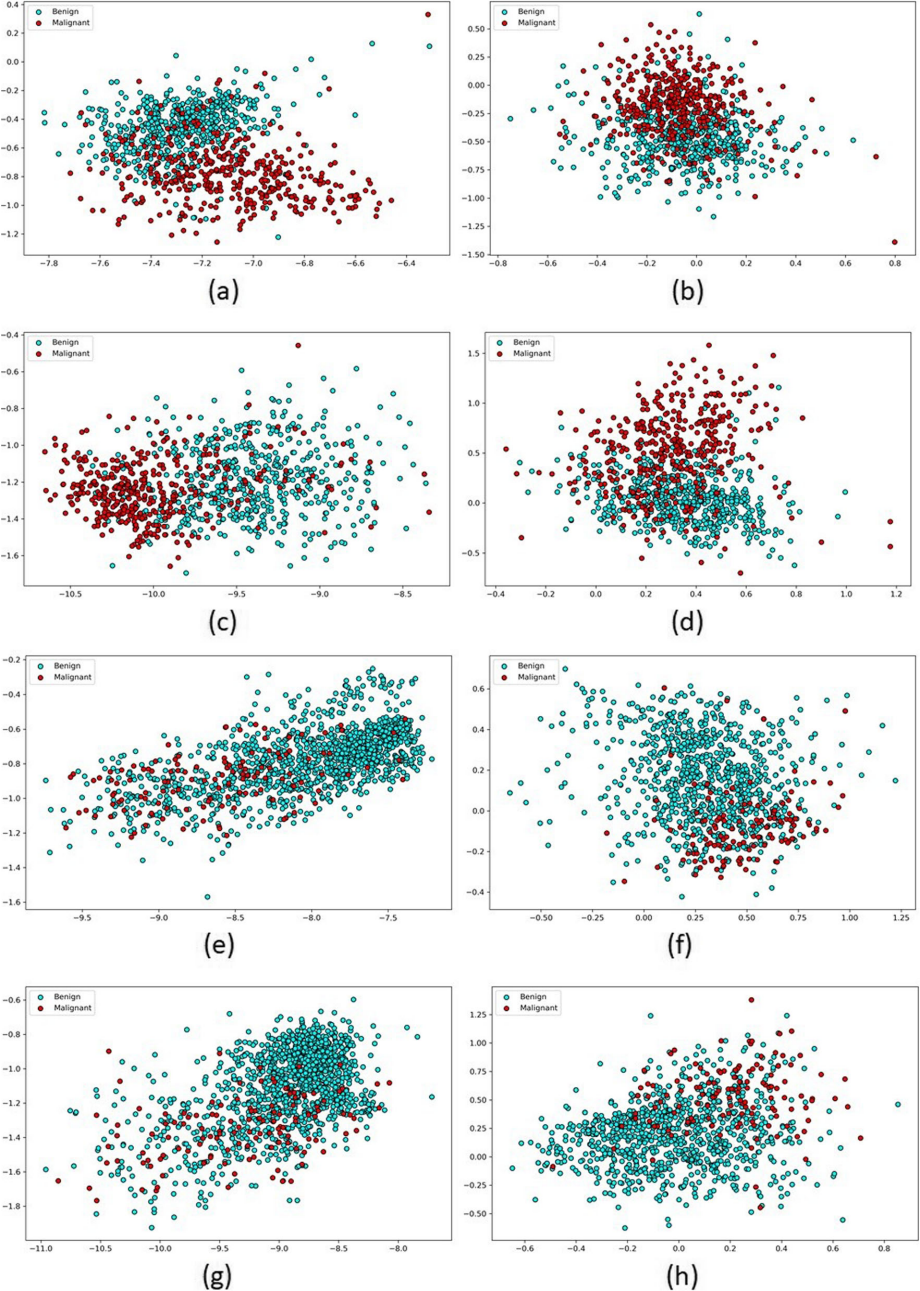
Mean vectors of reports in BERT and Word2Vec language spaces; left column: BERT space, right column: Word2Vec space; row 1: MR reports with MR language models, row 2: MR reports with MR-finetuned language models, row 3: US reports with US language models, row 4: US reports with US-finetuned language models

Given that BERT masked LM generates contextualized representation of word piece tokens rather than words, we plotted 2-dimensional compressed representations of malignant and benign reports for both domains (MR and US) for MR and US language models (Word2Vec and BERT) as well as cross-domain language models in Fig. 6. To generate report-wise representation, we computed mean of vectors of all tokens in the report just as we did for employing Random Forest classifier for prediction. Separation between malignant and benign reports indicates that classifier will be able to better distinguish between these two labels if trained in the given space. It is clear that malignant and benign reports are better separated for MR domain than they are for US domain. This can be attributed to severe class imbalance in US domain where minority class makes up only 10% of the data, as compared for MR domain. In addition, class separation is better in US Word2Vec language space than US BERT language space. Results in Table 3 confirm that discriminative model, e.g. Random Forest, performs better with US Word2Vec representation than US BERT representation. This trend can be attributed to larger training data requirement of BERT language model.

## Discussion

The study reports a successful transfer of language models from one domain to a similar domain in radiology and compares it to not performing adaptation. The core novelty of this whole pipeline is that only a limited domain-specific corpus is required to train the language models while performing the LM transfer. We showed that fine-tuning of the word-embedding models with similar domain adaptation (US → MR and MR → US), even for multi-institutional reports, provides more opportunity for semantic knowledge preservation for down-steam classification tasks compared to training the language model from scratch.

Our study shows a way to train language space model with limited number of reports by using cross-domain transfer. Our proposed pipeline automatically extracted binary labels (benign/malignant) for imaging dataset for HCC screening with high accuracy. Our proposed framework only needs a small subset of labeled exams to generate a large dataset of labeled exams. The labeled studies may be used for AI development and training, and such automated NLP methods will rapidly reduce the manual workload for creating labeled imaging datasets from hospital databases. The experimentation also shows that for the limited training dataset (in the order of thousands reports), context-independent distributional semantics models (Word2vec) performs better than the context-dependent transformer models (BERT). This surprising results could be due to small training corpus size for learning US and MR LM using BERT which has 110 M trainable parameters [13].

However, given the fact that collection of millions of task-specific radiology text reports, such as HCC screening, would be challenging from a single institution, training the transformer based models from scratch is often not a feasible option. Pre-trained transformer models with clinical dataset, such as BioBert [19], ClinicalBert [20], could be more adaptable but most important radiology-specific words may be missing from the LM space as they were trained on generic clinical datasets, and such words will be treated as word piece during fine-tuning in which they may loss the actual semantics.

This study lacks experimentation with the larger datasets since it is only bounded by the HCC screening reports. We believe that initial training of the transformer model with significantly large training data may help to outcome the Word2vec performance. In future, we plan to collect a larger multi-institutional radiology reports corpus and perform more experimentation with Bert and ELMo sequential embedding model.

## Conclusion

In this work, we explored the advantages of transfer learning for training language models between two distinct but similar domains. We chose US and MR reports for HCC screening as experimental domains. Our work involves multi-institutional data including data collected from Stanford Health Care (SHC) and Emory University Healthcare (EUH). We selected reports annotation with labels set of {‘malignant’, ‘benign’} as our downstream classification task for performance comparison. Our experiments clearly indicate that fine-tuning language models with similar domain adaptation (US → MR and MR → US) enables better preservation of semantic knowledge to improve classification performance as compared to training language model from scratch for each domain. This result is particularly interesting as similar domain transfer learning improves performance even with availability of relatively smaller corpus for language model training. We experimented with skipgram model training for word2vec language model as well as transformer model training for BERT language model. Given small training corpus, word2vec model performed much better than BERT model for downstream classification task. This trend indicates an important limitation of transformer based language modeling in clinical domain, i.e., requirement of extra large training corpus.

## Data Availability

The reports used in the study contains PHI and cannot be shared. But the training and validation reports can be obtained with data usage agreement. The trained models and code will be shared on first authors GitHub repository.

## Abbreviations

LM: Language model
US: Ultrasound
MR: Magnetic resonance
BERT: Bidirectional encoder representations from transformers
HCC: Hepatocellular carcinoma
